# Natural Language Processing Tools for Assessing Progress and Outcome of Two Veteran Populations: Cohort Study From a Novel Online Intervention for Posttraumatic Growth

**DOI:** 10.2196/17424

**Published:** 2020-09-23

**Authors:** Kim P Norman, Anita Govindjee, Seth R Norman, Michael Godoy, Kimberlie L Cerrone, Dustin W Kieschnick, William Kassler

**Affiliations:** 1 Young Adult and Family Center UCSF Weill Institute for Neurosciences University of California San Francisco, CA United States; 2 IBM Watson Health IBM Corporation Palo Alto, CA United States; 3 Tiatros, Inc San Francisco, CA United States

**Keywords:** natural language analysis, emotional tone, personality, values, PTSD, military sexual trauma, online interventions, internet-based cognitive behavioral therapy, narrative therapy, mindfulness

## Abstract

**Background:**

Over 100 million Americans lack affordable access to behavioral health care. Among these, military veterans are an especially vulnerable population. Military veterans require unique behavioral health services that can address military experiences and challenges transitioning to the civilian sector. Real-world programs to help veterans successfully transition to civilian life must build a sense of community, have the ability to scale, and be able to reach the many veterans who cannot or will not access care. Digitally based behavioral health initiatives have emerged within the past few years to improve this access to care. Our novel behavioral health intervention teaches mindfulness-based cognitive behavioral therapy and narrative therapy using peer support groups as guides, with human-facilitated asynchronous online discussions. Our study applies natural language processing (NLP) analytics to assess effectiveness of our online intervention in order to test whether NLP may provide insights and detect nuances of personal change and growth that are not currently captured by subjective symptom measures.

**Objective:**

This paper aims to study the value of NLP analytics in assessing progress and outcomes among combat veterans and military sexual assault survivors participating in novel online interventions for posttraumatic growth.

**Methods:**

IBM Watson and Linguistic Inquiry and Word Count tools were applied to the narrative writings of combat veterans and survivors of military sexual trauma who participated in novel online peer-supported group therapies for posttraumatic growth. Participants watched videos, practiced skills such as mindfulness meditation, told their stories through narrative writing, and participated in asynchronous, facilitated online discussions with peers. The writings, including online postings, by the 16 participants who completed the program were analyzed after completion of the program.

**Results:**

Our results suggest that NLP can provide valuable insights on shifts in personality traits, personal values, needs, and emotional tone in an evaluation of our novel online behavioral health interventions. Emotional tone analysis demonstrated significant decreases in fear and anxiety, sadness, and disgust, as well as increases in joy. Significant effects were found for personal values and needs, such as needing or desiring closeness and helping others, and for personality traits of openness, conscientiousness, extroversion, agreeableness, and neuroticism (ie, emotional range). Participants also demonstrated increases in authenticity and clout (confidence) of expression. NLP results were generally supported by qualitative observations and analysis, structured data, and course feedback.

**Conclusions:**

The aggregate of results in our study suggest that our behavioral health intervention was effective and that NLP can provide valuable insights on shifts in personality traits, personal values, and needs, as well as measure changes in emotional tone. NLP’s sensitivity to changes in emotional tone, values, and personality strengths suggests the efficacy of NLP as a leading indicator of treatment progress.

## Introduction

The lifetime risk of acquiring a mental illness diagnosis is 50%, yet over 100 million Americans lack affordable access to effective behavioral health care [1]. The economic costs of untreated mental illness are estimated in the hundreds of billions of dollars per year [2]. American military veterans are an especially vulnerable population. Up to 18% of Operation Enduring Freedom/Operation Iraqi Freedom veterans have posttraumatic stress disorder, and 25% have depression [3,4]. Furthermore, up to 41% of women and 4% of men are subjected to military sexual trauma, including sexual violence, sexual coercion, and severe and persistent sexual harassment [5]. It is estimated that 1.5 million of the 5.5 million veterans seen in US Department of Veterans Affairs (VA) hospitals in 2016 had a mental health diagnosis, and lack of access to mental health care is believed to contribute significantly to the high rate of suicide among US veterans, which tragically remains at 20 lives lost per day [6]. It is believed that at least half of veterans who need mental health care do not get the care they need, and more than half of those who would benefit from mental health care are unaware they need it [7].

With or without a mental health diagnosis, most returning veterans struggle with the transition to civilian life, suffering painful and sometimes debilitating symptoms even if they do not meet full criteria for a diagnosis [8]. One factor that exacerbates the difficulties of transition is the sense of profound loss created by being disconnected from the military community [9,10]. For example, veterans transition from almost never being alone to feeling all alone, leaving a life with a clearly established identity, culture, mission, purpose, and ever-present peer support to suddenly having to reinvent themselves. As a result, they tend to isolate [11] and consequently typically believe they are the only ones who feel this way. It can lead to loss of meaning and purpose, resulting from the stark contrast of going from high-intensity deployment, where every decision matters, to civilian life, where decisions seem much more trivial if they matter at all. The failure to address the special needs of veterans may explain why, sadly, real-world completion rates for in-person VA-approved therapies for posttraumatic stress disorder (PTSD) are 2% [12].

Real-world programs to help veterans successfully transition to civilian life must reach the many veterans who cannot or will not access care. These programs must have the ability to scale, as there is a shortage of services available to veterans, and they must address the loneliness that veterans experience while helping them reestablish their sense of mission, purpose, and connectedness. Therefore, successful interventions must also build a sense of community.

In an effort to expand access to care, digitally based behavioral health initiatives have emerged within the past few years. Most mental illnesses, including anxiety, depression, PTSD, and addiction, are effectively treated by cognitive behavioral therapy (CBT) [13]. The knowledge base of CBT best practices lends itself to scale. Internet delivery of CBT via therapist-mediated video chat and mixtures of online therapy sessions with email homework assignments are as effective as in-person therapies [14-18]. However, while improving reach, these approaches offer little or no scale [19]. Completely automated, open access, self-paced internet protocols offer scale, but have very low completion rates of around 1% [20,21]. Guided self-help, where clinicians offer support via brief phone calls or emails, have completion rates of 50% or higher and outcomes equal to in-person therapies [17]. Such interventions, however, rely heavily on the contacts between research associates and research participants, and the actual level of guidance provided is not usually discussed in the methodologies of these studies. Furthermore, few trauma-focused intervention protocols incorporate peer or social support, which has been identified as a protective factor against onset of PTSD and an integral part of recovery [22]. Additionally, an overlooked aspect of recovery from trauma is incorporation of military values. Personal accountability and group accountability are highly valued characteristics in military culture [23], yet rarely used in the treatment of veterans. Our novel behavioral health intervention teaches mindfulness-based CBT and narrative therapy using peer support groups as guides, with human-facilitated asynchronous online discussions. Our intervention attempts to address the aforementioned difficulties of online treatment—those of scale, lack of social support, and heavy reliance on clinical contact—while incorporating group accountability values that resonate with veterans.

Historically, the standard of assessing efficacy of a therapeutic intervention has been through administration of subjective symptom measures at various time points (usually pretreatment and posttreatment) [24-26]. These measures generate quantitative scores that become structured data [27]. Our online intervention, by virtue of administering written narrative and CBT exercises, generated large amounts of unstructured qualitative data that, in the course of successive weekly sessions, was longitudinal. Unstructured, qualitative data, while not generally the standard of intervention evaluation, may contain insights and nuances of personal change that are not captured by subjective symptom measures. Furthermore, data collection for posttreatment evaluation tends to suffer lower completion rates; thus, ground truth information about intervention effectiveness can be lost. The technological advancement of natural language processing (NLP) provides opportunities to generate insights in narrative- and CBT-based interventions by examining participants’ own language. In fact, use of NLP is a practice in the artificial intelligence (AI) field for attempting to cultivate emotional intelligence in conversational robotics [28]. Validity studies have shown that NLP reliably predicts personality characteristics, personal needs, and values, and monitors emotional tone and style of communication [29,30]. NLP offers the promise of enhancing scale by giving participants immediate feedback while providing clinicians with real-time, actionable observations. At the same time, NLP supplies researchers the data needed to assess the impact of the behavioral health intervention.

The purpose of our study was to evaluate the feasibility and utility of NLP in evaluating change in a small pilot study of 2 veteran populations who completed 2 novel online behavioral health interventions.

## Methods

### Participants

A total of 23 participants were recruited for 2 studies of veterans, the Next Mission (NM) program and Women Warriors (WW) program. A total of 13 participants enrolled in the 14-week NM program, and 10 enrolled in the 8-week WW program. Inclusion criteria for the study were: (1) must be a military veteran, (2) must be aged 18 or older, (3) must speak English, (4) must be able to access the internet regularly, and (5) for the WW program, must be a woman. In the NM program, 11 of the 13 (85%) participants were men, while the WW program consisted of all (10/10, 100%) female participants. In total, 16 participants completed the courses, 9 in the NM program and 7 in the WW program.

### Procedures

Participants were recruited via Facebook and LinkedIn ads and could sign up on a mobile, laptop, or desktop device of their choice. Recruitment was conducted over several months until enough participants were able to form cohorts for each course. The recruitment messages attempted to account for potential stigma by focusing on building resiliency skills and promoting posttraumatic growth rather than treating mental illness. Participants were informed that they would be helping others in their group while also getting help for themselves.

Participants were also incentivized by the opportunity to earn University of California college credits for their work, although only 3 of 16 (19%) participants took advantage. Participants understood that the program was fully compliant to the Health Insurance Portability and Accountability Act (HIPAA) and that they could choose to participate anonymously. However, they also had the option of revealing their identity to the group at any time they wished during the course. Both programs, which we also refer to as courses, were entitled “Stress, Resiliency and Post Traumatic Growth.” The constellation of the groups was generationally diverse and allowed older veterans to connect with younger veterans**.**

In each program, participants watched videos teaching principles of CBT, narrative therapy, behavioral activation, and mindfulness meditation. They submitted written homework, including journal entries and thought and mood logs, and participated in asynchronous discussions, all within a HIPAA-secure environment. Approximately 90 minutes of online class time and one hour per week of homework was completed by each participant. The asynchronous discussions were facilitated and monitored by a licensed, doctoral-level therapist. Of note, facilitators for the WW group were female. The content for the programs was created in various commercially available proprietary applications by faculty members in the Department of Psychiatry of a US University Medical Center and then assembled into the NM and WW programs and delivered on a commercially available proprietary platform.

### Measures

#### Linguistic Inquiry and Word Count

Linguistic Inquiry and Word Count (LIWC) is software designed to analyze word use within written text. It calculates the percentage of usage for sets of words, arranging them in 80 linguistic categories and generating output statistics for each of the categories [31,32]. LIWC uses a proprietary set of algorithms to produce 4 summary variables from the data: analytical thinking, clout, authenticity, and emotional tone [33]. Analytical thinking measures the degree to which words are used that suggest logical and analytic thinking patterns. Clout measures the characteristic of speaking from the perspective of high expertise and confidence. Authenticity measures the characteristic of more honest, personal, and self-disclosing language. Emotional tone measures the characteristic of expressing more positive emotions in an upbeat style and expressing less anxiety, sadness and hostility. LIWC has been shown across numerous studies to have internal consistency and validity [34].

#### IBM Watson Personality Insights

IBM Watson is a computer system that uses artificial intelligence to interpret unstructured data within natural language. IBM Watson’s Personality Insights is programmed to analyze natural language input and provide outputs of personality characteristics based on 3 models [35]: (1) the Big Five personality traits (openness, conscientiousness, extroversion, agreeability, and neuroticism) [36], (2) personal needs, and (3) personal values. Personal needs describe 12 dimensions of personality aspects that are likely to resonate with the participant [37]: excitement, harmony, curiosity, ideal, closeness, self-expression, liberty, love, practicality, stability, challenge, and structure. Personal values [38] describe 5 types of values that are important to an individual and likely to influence decision-making behavior [39]: helping others, tradition, life pleasure, achievement, and openness to change. Outputs generated from Personality Insights are likelihood ratios between 0 and 1 and are data driven, meaning an output for a variable is only produced if detected. A variable with a score above 0.5 indicates a greater than average tendency for the characteristic to be true [40]. Table 1 lists the definitions of detected levels of needs and values.

**Table 1:** Definitions of personal needs and values outputs.^a^

Characteristic		Description
**Needs**		
	Excitement	Emphasizes importance of getting out and living life, oriented toward having fun
	Harmony	Appreciation for other people, their viewpoints, or feelings
	Curiosity	Seeking discovery and desire for personal growth
	Ideal	Wanting perfection and seeking sense of community
	Closeness	Valuing connectedness with others
	Self-expression	Emphasizes the importance of expressing oneself and asserting individual identities
	Liberty	Have a desire for fashion and new things, as well as the need for escape and freedom
	Love	Valuing social contact, either one-to-one or one-to-many
	Practicality	Having a desire to accomplish things, a desire for skill and efficiency, including physical expression and experience
	Stability	Valuing sensibility, equivalence, and balance
	Challenge	Having desire to succeed and take on challenges
	Structure	Exhibit a grounded trait and a desire to hold things together. They need things to be well organized and under control
**Values**		
	Helping others	Showing concern for the welfare and interests of others
	Tradition	Emphasizes self-restriction, order, and resistance to change
	Life pleasure	Seek pleasure and sensuous gratification for themselves
	Achievement	Seek personal success for themselves
	Excitement	Emphasize independent action, thought, and feeling, as well as a readiness for new experiences

^a^Table content was taken and aggregated from IBM Watson Personality Insights [29].

#### IBM Watson Tone Analyzer

IBM Watson Tone Analyzer is an artificial intelligence–enabled text analysis tool produced by IBM Watson that uses AI to infer emotional tone through written text. Tone Analyzer is based on psycholinguistics theory and examines how day-to-day word usage correlates to manifest emotions [30], based on the International Survey on Emotion Antecedents and Reactions [41] data set. Tone Analyzer has undergone studies for consistency and validity against human analysis of emotion and shown no statistically significant difference between human-labelled emotion of text and Tone Analyzer analysis of emotional tone [30]. Tone Analyzer–generated data outputs of the 5 basic emotions enabled us to evaluate the presence of 5 basic emotions: anger, disgust, fear, joy, and sadness [42].

#### Qualitative Observations and Questionnaires

Participants reported feedback from the course and filled out structured subjective symptom questionnaires. Structured data were not analyzed quantitatively in comparison with NLP due to variations of sample size and low completion rates. As such, quantitative changes in structured data were observed qualitatively. Facilitators also reported subjective qualitative observations of participant progress. Structured measures included the Positive States of Mind Scale (PSOM) [43], Posttraumatic Growth Inventory (PTGI) [44], PTSD checklist for the Diagnostic and Statistical Manual of Mental Disorders, Fifth Edition (PCL-5) [45], Patient Health Questionnaires (PHQs) [46], Short Warwick-Edinburgh Mental Well-Being Scale (SWEMWS) [47], and the Brief Coping Orientation to Problems Experienced (COPE) [48].

### Statistical Analysis

Participants’ writing samples and online posts were analyzed using the LIWC [33], IBM Watson’s Personality Insights [49], and Tone Analyzer [50]. Detection scores (NLP outputs) were generated as outputs, upon which we conducted a pretest-posttest analysis of these language attributes at the beginning and end of each course. Analysis was conducted in IBM SPSS Statistics (version 26). Initially, repeated-measures 1-tailed *t* tests were run and effect sizes were calculated using Cohen *d*. However, to reduce the risk of type I statistical error from multiple *t* tests, variables were grouped into 4 multivariate categories: (1) LIWC variables, (2) personality trait variables, (3) personality values and needs variables, and (4) emotional tone variables. Omnibus testing in the form of repeated-measures multivariate analysis of variance (rMANOVA) was conducted with Bonferroni correction [51]. Effect sizes were calculated as partial η^2^ [52] and Cohen *d* effect sizes were retained from *t* tests.

## Results

### Overview

In total, 16 of the 23 (70%) participants completed the courses and 15 were able to have their unstructured data analyzed. Results of all rMANOVA analyses on NLP outputs are summarized in Table 2.

**Table 2:** Results of natural language analysis for personality traits, values, emotional tone, and Linguistic Inquiry and Word Count for treatment groups of Next Mission combat veterans and Women Warriors military sexual assault survivors.^a^

Characteristic		NM^b^ combat veterans (n=9)					WW^c^ military sexual assault survivors (n=6)			
		*F* test^d^ (*df*)	Cohen *d*	*P* value	Effect size (partial η^2^)	*F* test^d^ (*df*)		Cohen *d*	*P* value	Effect size (partial η^2^)
**Personality traits**										
	Openness	6.372 (1,8)	1.34	.04	0.443	9.740 (1,5)		1.47	.03	0.661
	Agreeableness	10.305 (1,8)	1.77	.01	0.777	12.211 (1,5)		1.68	.02	0.709
	Extroversion	12.446 (1,8)	1.52	.001	0.609	7.105 (1,5)		–1.58	.045	0.587
	Conscientiousness	27.923 (1,8)	–2.83	.001	0.563	1.929 (1,5)		–0.53	.22	0.278
	Emotional range	15.473 (1,8)	–1.74	.004	0.659	1.1014 (1,5)		0.58	.36	0.169
**Values and needs^e^**										
	Curiosity	3.227 (1,8)	0.84	.11	0.287	5.971 (1,5)		1.44	.06	0.544
	Harmony	7.787 (1,8)	1.42	.02	0.493	N/A^f^		N/A	N/A	N/A
	Structure	1.865 (1,8)	–0.44	.21	0.189	2.784 (1,5)		0.87	.16	0.358
	Closeness	17.672 (1,8)	2.23	.003	0.688	10.006 (1,5)		1.23	.03	0.667
	Stability	6.518 (1,8)	1.49	.03	0.449	N/A		N/A	N/A	N/A
	Helping others	21.715 (1,8)	2.06	.002	0.731	3.077 (1,5)		0.35	.14	0.381
	Excitement	23.635 (1,8)	2.34	.001	0.747	0.391 (1,5)		0.15	.56	0.072
	Life pleasure	18.926 (1,8)	1.92	.002	0.703	8.511 (1,5)		1.30	.03	0.630
	Tradition	9.005 (1,8)	–1.54	.02	0.530	1.238 (1,5)		0.12	.32	0.198
	Achievement	9.414 (1,8)	1.01	.02	0.541	7.294 (1,5)		1.28	.04	0.593
	Love	N/A	N/A	N/A	N/A	13.474 (1,5)		1.58	.01	0.729
	Ideal	N/A	N/A	N/A	N/A	3.173 (1,5)		1.03	.14	0.388
**Emotional tone**										
	Sadness	10.852 (1,8)	–1.01	.01	0.576	1.164 (1,6)		–0.20	.33	0.283
	Disgust	7.660 (1,8)	–1.25	.02	0.489	1.413 (1,5)		–0.74	.29	0.220
	Joy	11.017 (1,8)	1.81	.01	0.579	1.977 (1,5)		0.84	.22	0.283
	Fear	0.114 (1,8)	–0.17	.74	0.014	4.365 (1,5)		–0.80	.09	0.466
	Anger	0.542 (1,8)	–0.32	.48	0.063	0.518 (1,5)		–0.40	.50	0.094
**LIWC^g^ analysis**										
	Analytical thinking	0.229 (1,8)	0.20	.65	0.028	0.094 (1,5)		–0.12	.77	0.018
	Authenticity	7.326 (1,8)	0.92	.03	0.478	5.457 (1,5)		0.37	.07	0.522
	Clout	8.651 (1,8)	0.90	.02	0.520	7.920 (1,5)		1.47	.04	0.613
	Emotional tone	6.016 (1,8)	0.92	.04	0.429	4.597 (1,5)		1.17	.09	0.479

^a^N=15.

^b^NM: Next Mission.

^c^WW: Women Warriors.

^d^*F* values reported are from repeated-measures multivariate analysis of variance (rMANOVA), with effect sizes calculated using both Cohen *d* from repeated-measures *t* tests and partial η^2^ from rMANOVA tests. Significance calculated from rMANOVA test with Bonferroni correction.

^e^For each program group, 10 identified values were outputted by IBM Watson Personality Insights based on highest density, thus differ slightly between programs.

^f^N/A: not applicable.

^g^LIWC: Linguistic Inquiry and Word Count.

### NM Program

A total of 9 of the 13 (70%) Next Mission participants completed the course and were able to have their unstructured data analyzed. Results of analysis on NLP data were grouped into 4 outcome variable groups: (1) personality traits, (2) personal values and needs, (3) emotional tone, and (4) LIWC. Results from rMANOVA were reported as univariate analyses. Cohen *d* effect sizes from initial *t* tests were retained and reported.

#### Analysis of Personality Traits

Participants showed significant increases in openness (*F*_1,8_=6.37; *d*=1.34; *P*=.03; partial η^2^=0.44), agreeableness (*F*_1,8_=10.3; *d*=1.77; *P*=.01; partial η^2^=0.78), and extroversion (*F*_1,8_=12.46; *d*=1.52; *P*=.02; partial η^2^=0.609), while conscientiousness (*F*_1,8_=27.92; *d*=–2.83; *P*=.001; partial η^2^=0.563) and emotional range (*F*_1,8_=15.47; *d*=–1.74; *P*=.01; partial η^2^=0.659) significantly decreased.

#### Analysis of Personal Values and Needs

Participants showed significant increases in the values and needs of helping others (*F*_1,8_=21.72; *d*=1.98; *P*=.002; partial η^2^=0.73), closeness (*F*_1,8_=17.67; *d*=2.23; *P*=.003; partial η^2^=0.69), excitement (*F*_1,8_=23.63, *d*=2.34; *P*=.001; partial η^2^=0.75), life pleasure (*F*_1,8_=18.93; *d*=1.92; *P*=.01; partial η^2^=0.70), harmony (*F*_1,8_=7.79; *d*=1.42; *P*=.01; partial η^2^=0.493), achievement (*F*_1,8_=9.41; *d*=1.01; *P*=.05; partial η^2^=0.54), and stability (*F*_1,8_=6.52; *d*=1.98; *P*=.02; partial η^2^=0.45). Furthermore, participants showed a significant decrease in valuing tradition (*F*_1,8_=9.01; *d*=–1.53; *P*=.03; partial η^2^=0.53).

Curiosity (*F*_1,8_=3.23; *d*=0.84; *P*=.24; partial η^2^=0.28) increased but was not statistically significant. Likewise, structure (*F*_1,8_=1.87; *d*=–0.44; *P*=.10; partial η^2^=0.19) decreased but was not statistically significant.

#### Analysis of Emotional Tone

Participants showed significant decreases in sadness (*F*_1,8_=10.85; *d*=–1.01; *P*=.01; partial η^2^=0.58) and disgust (*F*_1,8_=7.66; *d*=–1.25; *P*=.02; partial η^2^=0.49), while joy significantly increased (*F*_1,8_=11.02; *d*=1.81; *P*=.01; partial η^2^=0.58).

Anger (*F*_1,8_=0.54, *d*=–0.32; *P*=.24; partial η^2^=0.06) and fear (*F*_1,8_=0.11; *d* =–0.17; *P*=.37; partial η^2^=0.01) decreased but were not statistically significant.

#### LIWC Analysis

The application of LIWC tools showed significant improvement in authenticity, clout, and emotional tone. Authenticity (*F*_1,8_=7.33; *d*=0.92; *P*=.01; partial η^2^=0.48) improved by 49%. Clout (*F*_1,8_=8.65; *d*=0.90; *P*=.01; partial η^2^=0.52) increased by 80%. Emotional tone (*F*_1,8_=6.02; *d*=0.92; *P*=.02; partial η^2^=0.43) improved by 51%. However, analytical thinking did not show significant change (*F*_1,8_=0.23; *d*=0.20; *P*=.65; partial η^2^=0.03).

#### Qualitative Observations

Observations were derived from structured data completed by 3 of the participants (results found in Table 2). The PTGI showed a general increase in the domains of relating to others, new possibilities, personal strength, and appreciation for life. The PSOM indicated no overall changes but did show an increase from all 3 participants in responsible caretaking. Course feedback included comments such as:

The site made me feel calm and cared for. It made me feel like people were looking out for me and that I had a network of resources and people if I needed.

I got my laughter back.

### WW Military Sexual Assault Survivors

In total, 7 of the 10 (70%) Women Warriors participants completed the course. Of these, 6 WW participants produced sufficient narrative to have their unstructured data analyzed. Results of analysis of unstructured data were grouped into 4 outcome variable groups: (1) personality traits, (2) personal values and needs, (3) emotional tone, and (4) LIWC. Results from rMANOVA were reported as univariate analyses. Cohen *d* effect sizes from initial *t* tests were retained and reported.

#### Analysis of Personality Traits

Participants showed significant increases in personality traits of openness (*F*_1,5_=9.74; *d*=1.47; *P*=.01; partial η^2^=0.66) and agreeableness (*F*_1,5_=12.21; *d*=1.68; *P*=.01; partial η^2^=0.71), while showing significant decreases in extraversion (*F*_1,5_=7.11; *d*=–1.58; *P*=.04; partial η^2^=0.59).

Conscientiousness (*F*_1,5_=1.93; *d*=–0.53; *P*=.22; partial η^2^=0.28) decreased and emotional range (*F*_1,5_=1.10; *d*=0.58; *P*=.36; partial η^2^=0.17) increased but these were not statistically significant.

#### Analysis of Personal Values and Needs

Participants showed significant increases in the personal values and needs of closeness (*F*_1,5_=10.01; *d*=1.23; *P*=.03; partial η^2^=0.67), love (*F*_1,5_=13.47; *d*=1.58; *P*=.01; partial η^2^=0.73), life pleasure (*F*_1,5_=8.51; *d*=1.30; *P*=.02; partial η^2^=0.63), curiosity (*F*_1,5_=5.97; *d*=1.44; *P*=.03; partial η^2^=0.54), and achievement (*F*_1,5_=13.47; *d*=1.28; *P*=.02; partial η^2^=0.73).

The values and needs domains of helping others (*F*_1,5_=3.10; *d*=0.35; *P*=.07; partial η^2^=0.38), stimulation (*F*_1,5_=0.39; *d*=0.15; *P*=.28; partial η^2^=0.07), tradition (*F*_1,5_=1.24; *d*=0.12; *P*=.16; partial η^2^=0.20), structure (*F*_1,5_=2.78; *d*=0.87; *P*=.08; partial η^2^=0.36), and idealism (*F*_1,5_=3.17; *d*=1.03; *P*=.07; partial η^2^=0.39) generally increased but were not statistically significant.

#### Analysis of Emotional Tone

None of the differences of emotional tone were statistically significant. Fear (*F*_1,5_=4.37; *d*=–0.8; *P*=.09; partial η^2^=0.47), disgust (*F*_1,5_=1.41; *d*=–0.74; *P*=.14; partial η^2^=0.22), and sadness (*F*_1,6_=1.16; *d*=–0.2; *P*=.16; partial η^2^=0.28) generally decreased, while joy (*F*_1,5_=1.98*; d*=0.84; *P*=.11; partial η^2^=0.28) generally increased.

#### LIWC Analysis

The application of LIWC tools showed significant improvement in clout (*F*_1,5_=7.92; *d*=1.47; *P*=.02; partial η^2^=0.61), which increased by 56%. Although not significant, emotional tone (*F*_1,5_=4.60; *d*=1.17; *P*=.08; partial η^2^=0.48) improved by 24%, and authenticity (*F*_1,5_=5.46; *d*=0.37; *P*=.07; partial η^2^=0.52) improved by 9.3%. Analytical thinking did not show significant change (*F*_1,5_=0.09; *d*=–0.12; *P*=.77; partial η^2^=0.02).

#### Qualitative Observations

Qualitative observations of structured data (results found in Table 3) were generally consistent with anecdotal reports from participants and facilitators. There were 7 participants who completed structured measures. The PHQ-15 showed general decreases in somatization levels, from moderately severe to minimal. The Brief COPE showed a general increase in acceptance, decreased denial, increases in getting support and asking advice, and increased feelings of hope (ie, not giving up). Of the 7 participants, 5 showed less anxiety (generalized anxiety disorder 7-item checklist) and 4 showed improvement in depression (PHQ-9). Results of the PCL-5 did not show overall improvement, but 2 of the 7 participants showed substantially reduced symptoms scores, while 3 others recorded no change. PTGI did not show overall improvement, but 4 participants showed a general improvement in relating to others and participation in life. Observations of the SWEMWS were mixed, as 2 participants improved and 5 remained the same. Course feedback included comments such as:

I believe [course facilitator] when you say WE WILL get to the place of balance and peace. I returned home and I feel better than I have in a long time. I still have challenges and I know it's a day to day journey, but I ACTUALLY feel stronger…I'm honestly still in shock. I didn't think recovery would ever be a word used to describe me, but now I'm believing it.

The good thing that came from this week's assignment was I found that I found support that I didn't realize that I had from sources I never would've thought.

[The course platform] made me feel calm and cared for. It made me feel like people were looking out for me and that I had a network of resources and people if I needed.

**Table 3:** Averages and change scores for treatment groups of Next Mission combat veterans and Women Warriors military sexual assault survivors.

Assessment		NM^a^ combat veterans (n=3)			WW^b^ military sexual assault survivors (n=7)		
		Pretest, mean	Posttest, mean	Difference score^c^, mean	Pretest, mean	Posttest, mean	Difference score^c^, mean
**Posttraumatic Growth Inventory (PTGI)**		47.3	58.7	11.4	66.6	60.6	–6.0
	PTGI-I: relating to others	10.7	17.0	6.3	21.0	18.6	–2.4
	PTGI-II: new possibilities	13.0	14.7	1.7	17.0	1.7	–15.3
	PTGI-III: personal strength	11.7	12.7	1.0	14.6	10.6	–4.0
	PTGI-IV: spiritual change	3.0	3.7	0.7	2.6	2.7	0.1
	PTGI-V: appreciation of life	9.0	10.7	1.7	8.6	8.7	0.1
SWEMWS^d^		N/A^e^	N/A	N/A	21.1	22.2	1.1
**Brief COPE^f^**		N/A	N/A	N/A	36.3	30.3	–6.0
	Active coping	N/A	N/A	N/A	3.1	2.6	–0.5
	Positive reframing	N/A	N/A	N/A	3.3	2.9	–0.4
	Plan	N/A	N/A	N/A	3.6	2	–1.6
	Emotional support	N/A	N/A	N/A	2.6	3	0.4
	Self-distraction	N/A	N/A	N/A	3.3	2.7	–0.6
	Vent	N/A	N/A	N/A	2.9	2.1	–0.8
	Behavioral disengagement	N/A	N/A	N/A	1.9	0.7	–1.2
	Acceptance	N/A	N/A	N/A	1.9	3.4	1.5
	Humor	N/A	N/A	N/A	2.9	1.9	–1.0
	Religion	N/A	N/A	N/A	3.1	2.3	–0.8
	Instrumental support	N/A	N/A	N/A	2.3	3.6	1.3
	Denial	N/A	N/A	N/A	0.9	0.4	–0.5
	Substance use	N/A	N/A	N/A	1.1	0.9	–0.2
	Self-blame	N/A	N/A	N/A	2.0	1.8	–0.2
PCL-5^g^		N/A	N/A	N/A	57.6	60.9	3.3
PHQ^h^-15		N/A	N/A	N/A	13.6	10.9	–2.7
PHQ-9		N/A	N/A	N/A	13.4	10.7	–2.7
GAD-7^i^		N/A	N/A	N/A	11.4	8.3	–3.1
**PSOMS^j^**		9.0	9.0	0.0	N/A	N/A	N/A
	Focused attention	1.7	1.7	0.0	N/A	N/A	N/A
	Productivity	2.0	1.3	–0.7	N/A	N/A	N/A
	Responsible caretaking	1.0	2.0	1.0	N/A	N/A	N/A
	Restful repose	0.7	1.3	0.6	N/A	N/A	N/A
	Sensuous pleasure	2.0	1.0	–1.0	N/A	N/A	N/A
	Sharing	1.7	1.7	0.0	N/A	N/A	N/A

^a^NM: Next Mission.

^b^WW: Women Warriors.

^c^Differences in scores were used for qualitative observation. Differences in scores were not calculated for significance due to variation in completion and sample size.

^d^SWEMWS: Short Warwick-Edinburgh Mental Well-Being Scale.

^e^N/A: not applicable.

^f^COPE: Coping Orientation to Problems Experienced.

^g^PCL-5: PTSD checklist for the Diagnostic and Statistical Manual of Mental Disorders, Fifth Edition.

^h^PHQ: Patient Health Questionnaire.

^i^GAD-7: generalized anxiety disorder 7-item checklist.

^j^PSOMS: Positive States of Mind Scale (n=10).

## Discussion

### Principal Findings

The purpose of our study was to evaluate the feasibility and utility of NLP in evaluating change in a small pilot study of 2 veteran populations who completed 2 novel online behavioral health interventions. Our aggregate results suggest that NLP can provide valuable insights on shifts in personality traits, personal values, and needs, as well as measure changes in emotional tone, in an evaluation of our novel online behavioral health interventions. The process of our participant recruitment suggests additional support for our findings. Recruitment was conducted over several months until critical mass was obtained for a cohort, with participants waiting variable amounts of time until start of the courses. Participants, regardless of waiting time, began the courses with similar symptom profiles. However, although our effect sizes are large, our small sample size must be considered in the interpretation of our results. Additionally, although it is reasonable to conclude that the large effect sizes seen are the result of our intervention, a limitation of our study was that we were unable to account for potential confounds by comparison with a control group. Thus, we cannot say with absolute certainty whether the effects seen were due to our intervention or additional factors. Future studies should seek to replicate the effects found in our pilot study with a control or comparison group, accounting for potential confounds.

In general, results of NLP mirrored qualitative reports and feedback from participants and facilitators on the efficacy of these interventions. Furthermore, our study suggests that NLP may detect participant change with a greater sensitivity than that of subjective symptom measures. We note that checklists do not tell stories—only stories do. A common clinical observation is the phenomenon in treatment of depression or anxiety wherein an individual’s loved ones or a clinician will often notice change in the individual before the individual become self-aware of the change [53,54]. In a similar manner, natural language analysis may provide an indication that change is occurring through the person’s own written text before there is meta-recognition of change and before such change is detectable using subjective symptom measures.

Findings such as decreased fear, sadness, disgust, and emotional range (neuroticism) with increased joy are consistent with posttraumatic growth and specifically tend to be associated with resilience. A common phenomenon in combat veterans is survivor’s guilt, more conventionally termed moral injury [55]. The nature of combat is such that it lends itself to situations that are morally ambiguous or unclear, potentially culminating in significant moral conflict after the fact. The constructs of moral injury and PTSD overlap, although they have characteristics that differentiate them [56]. A key component of moral injury is the presence of guilt or shame as a result of a real or perceived transgression [55]. From an evolutionary psychology perspective on emotions, guilt and shame are considered derivatives of the emotion of disgust [57]. Furthermore, there is evidence that elevated levels of disgust are associated with increases in intrusive thoughts of trauma [58] and with posttraumatic stress symptoms in general [59]. There is further evidence to suggest that inclusion in a supportive community of individuals with shared experiences is known to reduce feelings of guilt and shame while increasing feelings of acceptance and belonging [60]. Hence, the results of our study may suggest that reductions in the emotion of disgust could correlate with reductions in guilt and shame and could subsequently correspond to symptom change in PTSD. Although there is indication of this effect from our findings, further exploration and replication is required.

A noteworthy finding of our study is revealed by the consistent increases in closeness, love, and life pleasure in WW military sexual assault survivors. PTSD research indicates that these domains are severely impacted by trauma in general and sexual assault in particular [61]. In fact, one of the main treatment targets for cognitive processing therapy of PTSD is reduced ability to trust [62], which is associated with negatively impacting closeness and intimacy. In sexual assault survivors, intimacy and closeness can become significantly more difficult in the wake of the trauma, especially when combined with other sequelae of PTSD symptoms [63,64]. The findings of our study suggest that this intervention is helpful in fostering an enhanced ability to become close and intimate, and indeed to rely on others for social support. A potential explanation is that the medium in which survivors of trauma interact, asynchronously through the web, provides a sense of psychological safety that enables taking healthy risks to interact with others. In other words, the interaction can feel safe because it removes potential external and environmental triggers, which can be physiologically and cognitively destabilizing in the moment. Additionally, knowing that social support is a significant protective factor for PTSD [22], the participants in our study could find additional psychological safety in knowing that the other members of the group had been through a similar experience. In fact, it is already established that support groups can be efficacious [65], but in populations with PTSD, avoidance can be a significant obstacle to treatment [66]. It suggests that a slow gradual approach to social interaction through a technological medium can produce significant changes in the ability to experience closeness and intimacy, as well as in needing and valuing others. Finally, we believe there is therapeutic value inherent in the task of writing about one’s adverse experiences [67].

There is tentative evidence to suggest that the personality trait of conscientiousness is related to hypervigilance in decision making [68]. Thus, our findings may suggest that hypervigilance, a cardinal symptom of PTSD, may reduce in tandem with reduced conscientiousness. It is quite possible that on subjective symptom measures, individuals may not realize reductions in hypervigilance due to their identification as someone who is “always on guard,” a concept that has overlap and is consistent with military cultural values. However, additional research is needed to establish a clear association between the constructs of conscientiousness and hypervigilance.

A possible explanation for increased need for stability (eg, a consumer who consistently likes the same choice in a product, not a variety of choices) is that as veterans connect and create community with each other, there may be a reflection back to shared military values. Stability and regimen are a hallmark of military culture [23]. In fact, one participant reported via course feedback that “the good thing that came from this week's assignment was I found that I found support that I didn't realize that I had from sources I never would've thought.”

Further studies are needed and are underway to determine whether a higher rate of completion of subjective symptom measures will correspond to changes found with NLP. These studies are also needed to determine whether NLP changes will accurately predict future changes in subjective symptom measures.

We recognize that with the attributes being measured by these tools, aggregating data is not always the best way to assess whether the impact of an intervention on an individual is positive, negative, or neutral. For example, authenticity and confidence in expression are generally understood in terms of “the more the better,” whereas for attributes like conscientiousness, the desired outcome would be more for someone who is irresponsible and less for someone who is overly obsessive or rigid. Future research should focus on evaluating these online behavioral health interventions with larger samples across different populations while also measuring effectiveness in comparing natural language analytics to conventional evaluation methods.

A limitation of our study is that the generalizability is impacted by small sample sizes and the lack of a control group. Thus, we are unable to confirm whether results are due to the online intervention or another unidentified factor or set of factors. Furthermore, despite a growing number of validation studies, NLP is not widely accepted or implemented as a reliable indicator of therapeutic change. Thus, further validation studies that establish convergent and discriminant validity with therapeutic outcomes are warranted. An additional limitation of our study is that we were unable to statistically compare outputs from NLP to validated structured measures. The extant validation literature suggests that attempts at finding convergence of NLP with self-report measures of personality have produced mixed results. This could be due to the fact that NLP tends to measure both latent and explicit emotional tone and personality, whereas self-report measures are solely reliant on the perception of the individual. As a result, both methods have accrued criticism for being affected by bias [69]. However, the two methods may not measure the same construct, but different constructs from differing perspectives. Additionally, although there is initial evidence that NLP outputs can be predictors of consumer behavior [70], there is not yet certainty that those variables are predictors of therapeutic or behavioral change or that they impact psychosocial outcomes. On the other hand, symptom reduction measures have a better representation and acceptance as predictors of therapeutic and behavioral change within the extant literature. Thus, future studies should attempt to replicate our findings using NLP with larger sample sizes of veterans and construct a study design in such a way that NLP can be evaluated as a predictor of psychosocial outcomes.

### Conclusions and Implications

Our interventions were impactful on attributes detected in writing, and the results of NLP provided tentative yet potentially valuable and provocative insights. By quantifying and aggregating these attributes, we have gained insights about which areas of emotional functioning are responsive to our intervention. By looking at individual analyses, we can readily see how each participant is progressing, for example by noting reduced emotional expression in someone who was emotionally dysregulated and increased emotional expression in another participant who was initially emotionally constricted. The use of natural language analytics tools opens up a completely new area of scientific inquiry. We are getting closer to entirely replicating what happens in in-person psychotherapy. We can now provide the benefit of both symptom checklists and patient narratives to expert clinicians who know how to interpret the data for clinical decision making and to researchers who can determine the impact and value of any given behavioral health intervention. We believe that using AI powered by natural language analytics will enable the creation of effective therapy bots that will assist facilitators and sustain participant engagement, as this intervention is scaled to make it accessible to everyone, anytime, anywhere. We also believe that using NLP applied to behavioral health interventions and other clinical situations creates an entirely new field of medical informatics.

## Abbreviations

AI: artificial intelligence
CBT: cognitive behavioral therapy
COPE: Coping Orientation to Problems Experienced
HIPAA: Health Insurance Portability and Accountability Act
LIWC: Linguistic Inquiry and Word Count
NLP: natural language processing
NM: Next Mission
PCL-5: PTSD checklist for the Diagnostic and Statistical Manual of Mental Disorders, Fifth Edition
PHQ: Patient Health Questionnaire
PSOM: Positive States of Mind Scale
PTGI: Posttraumatic Growth Inventory
PTSD: posttraumatic stress disorder
rMANOVA: repeated-measures multivariate analysis of variance
SWEMWS: Short Warwick-Edinburgh Mental Well-Being Scale
VA: US Department of Veterans Affairs
WW: Women Warriors
